# A Ketogenic Low-Carbohydrate High-Fat Diet Increases LDL Cholesterol in Healthy, Young, Normal-Weight Women: A Randomized Controlled Feeding Trial

**DOI:** 10.3390/nu13030814

**Published:** 2021-03-02

**Authors:** Jonas Burén, Madelene Ericsson, Nágila Raquel Teixeira Damasceno, Anna Sjödin

**Affiliations:** 1 Department of Food, Nutrition and Culinary Science, Umeå University, 90187 Umeå, Sweden; anna.sjodin@umu.se; 2 Department of Public Health and Clinical Medicine, Medicine, Umeå University, 90187 Umeå, Sweden; 3 Department of Medical Biosciences, Physiological Chemistry, Umeå University, 90187 Umeå, Sweden; madelene.ericsson@umu.se; 4 Umeå Centre for Molecular Medicine, Umeå University, 90187 Umeå, Sweden; 5 Department of Nutrition, School of Public Health, University of Sao Paulo, 05508-060 Sao Paulo, Brazil; nagila@usp.br

**Keywords:** cardiovascular disease, lipoproteins, saturated fatty acids, diet intervention, female, dietary fat

## Abstract

Ketogenic low-carbohydrate high-fat (LCHF) diets are popular among young, healthy, normal-weight individuals for various reasons. We aimed to investigate the effect of a ketogenic LCHF diet on low-density lipoprotein (LDL) cholesterol (primary outcome), LDL cholesterol subfractions and conventional cardiovascular risk factors in the blood of healthy, young, and normal-weight women. The study was a randomized, controlled, feeding trial with crossover design. Twenty-four women were assigned to a 4 week ketogenic LCHF diet (4% carbohydrates; 77% fat; 19% protein) followed by a 4 week National Food Agency recommended control diet (44% carbohydrates; 33% fat; 19% protein), or the reverse sequence due to the crossover design. Treatment periods were separated by a 15 week washout period. Seventeen women completed the study and treatment effects were evaluated using mixed models. The LCHF diet increased LDL cholesterol in every woman with a treatment effect of 1.82 mM (*p* < 0.001). In addition, Apolipoprotein B-100 (ApoB), small, dense LDL cholesterol as well as large, buoyant LDL cholesterol increased (*p* < 0.001, *p* < 0.01, and *p* < 0.001, respectively). The data suggest that feeding healthy, young, normal-weight women a ketogenic LCHF diet induces a deleterious blood lipid profile. The elevated LDL cholesterol should be a cause for concern in young, healthy, normal-weight women following this kind of LCHF diet.

## 1. Introduction

There is a resurgence of interest in ketogenic diets [1]. Nutritional ketosis usually occurs when restricting the carbohydrate intake to below 20–50 g per day [2], but there are individual variations [3]. The low amount of carbohydrate in these diets is usually compensated by a high fat content whereas protein is moderate. Thus, under isocaloric conditions the term “low-carbohydrate high-fat” (LCHF) is more appropriate [4]. Alongside its well-documented weight-reducing effects [5,6], recent studies have also shown a beneficial impact of ketogenic LCHF diets on cardiovascular risk factors [5]. However, the typically high intake of saturated fatty acids and low intake of dietary fiber in LCHF dietary regimes increases low-density lipoprotein (LDL) cholesterol, a risk factor for coronary heart disease [7,8]. In addition, findings from epidemiological studies support a specific role for small, dense LDL particles in the etiology of atherosclerosis [9,10,11]. However, there is no consensus from recent meta-analyses regarding the relationship between dietary saturated fat and the risk of cardiovascular disease (CVD) [12,13,14,15]. Hence, there are conflicting data regarding LCHF diets and cardiovascular health [1].

Today, many people consider low-carbohydrate diets as generally healthy, and low-carbohydrate and ketogenic diets are very popular diets globally [16]. In the light of the foregoing, and the increasing interest in ketogenic LCHF diets in new and widely different areas such as exercise [17], cognitive function [18] and gut microbiota [19], it is not surprising that the diet has also become popular among healthy, normal-weight individuals. In a large Swedish population-based sample where diet intake has been monitored repeatedly in the same individuals, follow-up data from the last 10-year period (until 2014) show less carbohydrate and higher protein and fat intakes [20]. Interestingly, recent food survey data from Swedish female university students suggest that the observed national transition from carbohydrate to fat intake persists, at least among individuals interested in food and nutrition [21]. Notably, ketogenic LCHF diet interventions in healthy, normal-weight humans that measured serum biomarkers for CVD are dominated by men. There is a single study with only women [22] and, although some of the other studies [23,24,25] include both healthy women and men, data are disaggregated by sex in only one intervention [23]. There are well-known sex-specific differences in metabolic physiological systems, caused by the inherently different biology of the two sexes. It is therefore possible that women and men respond differently to a diet regime. It is noteworthy that all previous studies with female participants [22,23,24,25] lack a National Food Agency (NFA) recommended control diet, and instead use participants’ habitual diets as the control diet.

Here we report the results of a four-week, randomized and highly controlled feeding trial with crossover design in healthy, normal-weight women following a ketogenic LCHF diet and a Swedish National Food Agency recommended control diet. The objective of the present study was to explore the effects of a ketogenic LCHF diet on LDL cholesterol (primary outcome), LDL subfractions, and conventional cardiovascular risk factors in blood.

## 2. Materials and Methods

### 2.1. Study and Design Overview

**Figure 1:**
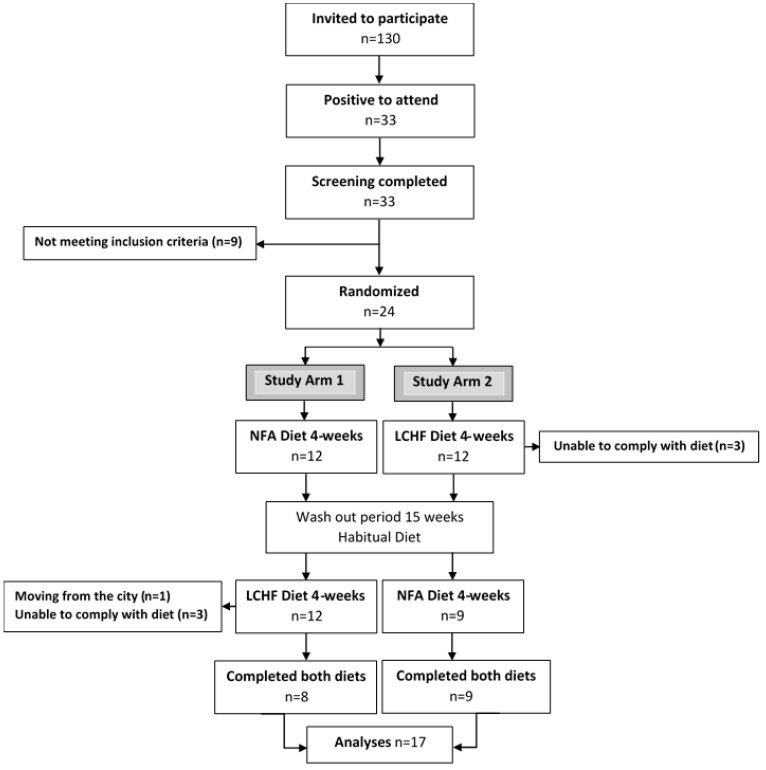
Consort flow chart. Adapted from [27]. NFA Diet, a National Food Agency recommended control diet; LCHF Diet, a ketogenic low-carbohydrate high-fat diet.

The trial has a cross-over design with two four-week diet periods separated by a 15 week washout period, with participants’ habitual diets. Healthy, young, normal-weight women were randomized to receive either a ketogenic LCHF diet or a Swedish National Food Agency recommended control diet for four weeks (Figure 1). After the washout period the groups were reversed and each woman received the other diet for four weeks. In order to evaluate diet-specific effects per se the study was designed to keep participant’s weight and physical activity stable. The primary outcome for the trial is LDL cholesterol. All other outcomes are secondary. Full details of the study protocol have been published elsewhere [26]. The clinical trial was registered at www.clinicaltrials.gov (accessed on 1 March 2021) as NTC02417350.

### 2.2. Ethics

The study was conducted according to the guidelines in the Declaration of Helsinki and all procedures were approved by the ethical review board at Umeå University, Umeå, Sweden (Dnr 2014-361-31M and Dnr 2015-45-32M).

### 2.3. Eligibility Criteria, Participants, and Study Setting

Inclusion and exclusion criteria have been described elsewhere [25]. In brief, healthy, young (18–30 years), female dietetics students were recruited at Umea University, Umea, Sweden. Participants were enrolled by a research nurse and given a specific consecutive numerical code.

Altogether 33 subjects were screened, of which 24 fulfilled the inclusion criteria, leaving 24 subjects to be randomized (Figure 1). Seven subjects dropped out during the study. One subject moved from the city, five withdrew during the first week eating the ketogenic LCHF diet with side effects such as headache, fatigue and nausea (*n* = 4), and feeling depressed (*n* = 1). A further subject dropped out after 15 days with the ketogenic LCHF diet with persistent feelings of fatigue, nausea, and abdominal pain. Altogether 17 subjects completed the full study. The baseline characteristics of these 17 subjects are presented in Table 1.

The study was conducted in Umeå, a small town in northern Sweden (population approximately 85,000). Measurements were taken at Umeå University and at University Hospital of Umeå.

### 2.4. Allocation and Blinding

Eligible participants were randomly allocated in 1:1 ratio to one of the two study arms (Figure 1). Minimization was used for the allocation sequence in order to minimize imbalance of the factor weekly training sessions [28,29]. The sequence of the two diets in study arm 1 and study arm 2, respectively, was decided by a research nurse who did not participate in the analysis of data. Consequently, the researchers were blinded to group allocation until the data were analyzed and the code key was broken.

### 2.5. Study Diets

A thorough description of the food, food handling, and photographs of the food can be found in the study protocol [26] and in [27]. Briefly, participants were provided with all food and not allowed to drink anything but water, coffee, and tea. In the LCHF diet the daily intake of carbohydrates should not exceed 25 g, excluding fiber. Carbohydrates were replaced by fat (77% of daily energy intake (E%)), with a high proportion of saturated fatty acids (33E%). The LCHF diet was based on meat, fish and seafood, eggs, high-fat dairy, coconut fat, olive oil, raspberries, avocado, nuts, and above ground vegetables such as broccoli and aubergine. The composition of the control diet was based on current dietary guidelines [30]. The control diet included plenty of vegetables, fruit and berries, fish and chicken, vegetable oils, high-fiber products, and low-fat dairy. Participants were instructed by a research nurse to consume all food provided, and to be weight stable. If they started to lose weight, they were instructed to eat extra pre-prepared snacks with the same macronutrient distribution as the respective study diet. There was no use of supplements and no intake of artificial sweeteners during the diet interventions.

### 2.6. Dietary Adherence and Physical Activity

In order to evaluate dietary adherence, participants were asked to make a daily note of any deviations from the dietary protocol. A previous publication describes the results regarding dietary intake, calculated from participants’ daily notes on deviations from planned food, and levels of ketone bodies in urine and blood [27]. The results showed excellent compliance to the planned diets and urinary ketosis was detectable after a maximum of four days and thereafter throughout the LCHF diet intervention (Ketostix, Bayer, Basel, Switzerland) (Supplemental Figure S1), and all women had blood β-hydroxybutyrate concentrations >0.5 mM at the end of the LCHF diet intervention. Participants also maintained their habitual physical activity levels [27].

During the intervention, weight was reduced during both the LCHF (~3 kg) and the control (~1 kg) diet [27].

### 2.7. Blood Sample Collection

Fasting blood samples were obtained at a Clinical Research Center by a research nurse according to a standard operating protocol as described earlier [27].

### 2.8. Biochemical Analysis

Glucose, insulin, plasma total cholesterol (TC), LDL cholesterol, HDL cholesterol, triacylglycerol (TG), Apolipoprotein A-I (ApoA-I), and Apolipoprotein B-100 (ApoB) were assayed within two hours from blood drawing at an accredited medical lab (Department of Clinical Chemistry, Umeå University Hospital) on a Cobas 8000 modular analyzer series (Roche Diagnostics International, Rotkreuz, Switzerland).

Fasting serum β-hydroxybutyrate concentrations were enzymatically determined in triplicate using a colorimetric assay kit (Cayman Chemical Company, Ann Arbor, MI, USA).

LDL subclasses were analyzed in serum by gradient electrophoresis in a polyacrylamide gel tube by using the Lipoprint LDL subfraction analysis system from Quantimetrix (Redondo Beach, CA, USA) according to manufacturer instructions [31]. This commercial and validated method measures subfractions of LDL (LDL 1–7) as percentages. Subclasses 1 and 2 were categorized into large, buoyant LDL, and subclasses 3 to 7 into small, dense LDL. To establish the concentration of cholesterol in each subfraction, the percentage was adjusted by total cholesterol and the final results are expressed in mg/dl. The peak particle diameter for phenotype A was set to >26.5 nm, and ≤26.5 nm for phenotype B.

### 2.9. Statistical Analyses and Power Calculations

Blood data, with and without adjustment for relative weight change, were analyzed using a mixed model, with post measurements of each period as the dependent variable. Period baseline measurements, and diet were included as fixed effects and subject was included as a random effect. In addition, subject-averaged period baseline values were included as fixed covariates, to avoid cross-level bias [32]. Analyses were performed using R version 3.5.3 (R Foundation for Statistical Computing, Vienna, Austria). The mixed model was fitted using the R function *lme* from the *nlme* package [33]. The impact of carry-over effects was investigated using visual assessments of line plots for the outcome variables at both individual and aggregated level for the two study arms, and by including the order of intervention as a fixed effect in the models.

As described in the study protocol [26], a power analysis based on the expected changes in LDL cholesterol was performed for sample size estimation. It was estimated that 16 participants would yield a power of 80% in detecting a significant difference between diets, given an α-error of 0.05. Considering a dropout rate of up to one third, we needed to recruit 24 participants.

## 3. Results

### 3.1. Primary Outcome

Eating LCHF diet for four weeks induced a higher LDL cholesterol compared to control diet (1.82 mM, 95% confidence interval (CI): [1.24, 2.39], *p* < 0.001, Table 2).

**Figure 2:**
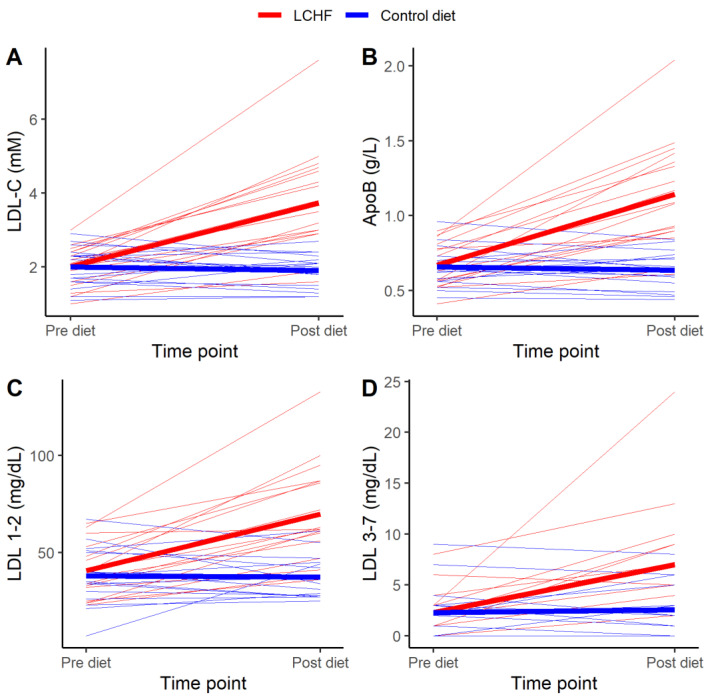
Individual (*n* = 17) changes of (**A**) low-density lipoprotein cholesterol (LDL-C), (**B**) Apolipoprotein B-100 (ApoB), (**C**) large, buoyant LDL (LDL 1–2), and (**D**) small, dense LDL (LDL 3–7). Pre diet (i.e., immediately before diet intervention) and Post diet (after four week diet intervention). The ketogenic low carbohydrate high fat diet (LCHF) is indicated with red lines, and the Control diet is indicated with blue lines. Thick lines represent mean values for the respective diets.

LDL cholesterol increased in every participant with a median endpoint LDL cholesterol of 3.50 [2.90–4.65] mM. Individual responses in LDL cholesterol to the LCHF diet are shown in Figure 2A.

LDL cholesterol remained higher in the LCHF group, when analysis was adjusted for relative weight change (Supplemental Table S1).

### 3.2. Secondary Outcomes

As seen in Table 2, eating LCHF for four weeks induced statistically significant differences in all secondary outcomes, except for mean LDL size, compared to control diet. LCHF induced statistically significantly lower values of glucose and insulin, and statistically significantly higher values of large, buoyant LDL (LDL 1–2), small, dense LDL (LDL 3–7), TG, TC, HDL cholesterol, non-HDL cholesterol, ApoB, ApoA-I, TC/HDL, ApoB/ApoA-I, and LDL/HDL compared to the control diet. Individual responses in ApoB, large, buoyant LDL and small, dense LDL to the LCHF diet are shown in Figure 2B, C and D, respectively. Except for glucose, the treatment effects on these blood parameters remained statistically significant when data were adjusted for relative weight change (Supplemental Table S1).

Before the ketogenic LCHF diet intervention, 16 participants were classified as pattern A (dominated by large buoyant LDL), and one participant as pattern B (dominated by small, dense LDL). After the LCHF diet intervention the one participant with pattern B switched to pattern A. Before the control diet intervention, all participants were classified as pattern A and the diet did not change this.

## 4. Discussion

This highly controlled feeding trial showed that feeding healthy, young, normal-weight women a ketogenic LCHF diet, rich in SFA and low in dietary fiber, for four weeks induced profound and widespread alterations in blood lipids. Plasma concentrations of LDL cholesterol (primary outcome) increased in every participant with a treatment effect of 1.82 mM. Considering the direct, graded relationship between LDL cholesterol concentration and the incidence of CVD in randomized controlled trials, prospective cohort studies, and Mendelian randomization studies [34,35], the results should be paid great attention by healthy, normal-weight women who perceive ketogenic diets to be a healthy nutritional option.

When, as in our study, the dietary intake of carbohydrates is restricted, the endocrine response will increase the ability to use dietary fat, and fatty acids released from the adipose tissue, for energy production. Fasting glucose and insulin will decrease, and increased levels of adrenaline and glucagon will increase fatty acid supply to the liver enhancing gluconeogenesis. Increased fatty acid oxidation leads to an increased production of ketone bodies (acetoacetate, beta-hydroxybutyrate, and acetone). The water-soluble ketone bodies can then enter the circulation and be used by tissues normally requiring glucose, particularly the brain. Thus, in this state of nutritional ketosis, the body has shifted from an insulin-mediated glucose-dependent condition to one with an increased ability to use fat as fuel [3].

Only a handful of studies have measured lipid status in healthy, young, normal-weight individuals following a ketogenic LCHF diet for more than two weeks [23,24,25,36,37,38]. All, except one study [37], report rather small, but nonetheless statistically significant, deteriorations in total and LDL cholesterol following this diet. However, none of these studies reports such profound increase in total and LDL cholesterol as the present feeding trial. This is probably explained by the strict protocol [26] of the current trial where participants were provided with all food consumed. Data from the diet and exercise logbooks, ketone body measurements in urine and blood, as well as blood glucose and insulin analyses indicate excellent compliance with the prescribed diets and maintained physical activity. This suggests that our strategies to improve adherence were successful.

Of the few studies in healthy, young, normal-weight individuals following a ketogenic LCHF diet for more than two weeks, one was performed with women [22], three were performed on men exclusively [36,37,38], whereas three were performed on mixed groups of men and women [23,24,25]. The recently published three week-long single arm study by Valsdottir et al. [22] reported changes in blood lipids that were in line with our findings. In their study, total cholesterol increased by 1.2 mM and LDL cholesterol increased by 0.9 mM after three weeks on a ketogenic LCHF diet. Another recently published study by Retterstol [24] contained a majority of women. In that study with parallel group design, three weeks of a LCHF diet consumed *ad libitum* increased LDL cholesterol with 0.9 mM. These reported findings of increased LDL cholesterol are much lower than the treatment effect of 1.82 mM in our present study. In Retterstol’s study the LCHF diet was self-selected, thus the type fatty acid intake, as well as proportions of fat and protein intake differed among participants. Furthermore, although the participants were instructed to adhere to the guidelines in Dr. Atkins´ New Diet Revolution [39] with the purpose of limiting carbohydrate intake, measurements of ketosis, an important indicator of compliance, were lacking. One must also keep in mind that both Valsdottirs’ and Retterstols’ studies were designed with their participants´ habitual diet as the control diet. It is therefore difficult to fully compare the results of their study with our randomized controlled feeding trial with a NFA recommended control diet.

In the current trial, we also determined the distribution of LDL subclasses using gradient gel electrophoresis. Small, dense LDL particles are considered more atherogenic than large particles, as some previous epidemiological studies demonstrated that individuals with predominantly small LDL particles had greater CVD risk than those with predominantly large LDL particles [9,10,11]. However, it has also been suggested that all LDL particles are atherogenic irrespective of LDL particle size [40]. In line with this, familial hypercholesterolemia patients with early atherosclerosis have a predominance of large, buoyant LDL particles [41]. It is notable that the recent expert consensus recommendations for dyslipidemia testing [42] state that patient treatment should focus on a reduction of the number (concentration) of LDL particles, regardless of particle size. The effect of ketogenic diet on small, dense LDL levels in healthy, normal-weight subjects has only been reported in one study, Sharman et al. [37], where all participants were men. They found that men classified as pattern A responded differently to the ketogenic diet, as compared to men classified as pattern B. They reported that LCHF induced no significant changes in the percentage of any LDL subclasses or mean particle size in pattern A subjects. However, in pattern B subjects, LCHF induced a statistically significant increase of LDL-1, a decrease of LDL-2 and 3 subclasses, as well as an increase in mean LDL particle diameter. In contrast to Sharman’s study of healthy men, we found an increase of large, buoyant LDL (LDL subclass 1 and 2) in every participant eating ketogenic LCHF diet for four weeks, with a treatment effect of 31.56 mg/dL, despite dominance of pattern A participants. In addition, every participant classified as pattern A also showed increased concentration of small, dense LDL. However, this change was not enough to shift the pattern from A to B. Notably, in line with earlier findings by Sharman and colleagues, our pattern B participant was the only one that responded to the ketogenic LCHF diet with a decreased concentration of small, dense LDL. Overall, the treatment effect of LCHF diet on small, dense LDL in our trial was 4.51 mg/dL. In addition, we report an extensive increase in ApoB indicating an increase of the total number of atherogenic lipoproteins in the circulation [43]. Taken together, the current ketogenic diet rich in SFA promotes changes in total LDL, small dense LDL, and ApoB that contributes to atherogenic risk in normal-weight women. Whether ketogenic LCHF diets with a different fatty acid composition would induce different effects in normal-weight women remains to be investigated in future studies.

Beside LDL cholesterol, data for TC, HDL cholesterol, non-HDL cholesterol and TG make up the primary lipid panel for hyperlipidemia diagnosis and cardiovascular risk estimation [44]. TC represents the cholesterol content in all circulating lipoproteins and a diet-induced decrease in TC correlates (as for LDL cholesterol) well with the degree of reduction in CVD [13]. Non-HDL cholesterol, on the other hand, represents the cholesterol in all atherogenic particles. Thus, in contrast to LDL cholesterol, non-HDL cholesterol takes into account the atherogenic potential of remnant lipoproteins [44]. Meta-analysis data from epidemiological studies suggest that non-HDL cholesterol is at least equally good as LDL cholesterol as a predictor of cardiovascular risk in the general population [45]. Our reported changes in both TC and non-HDL cholesterol following the ketogenic LCHF diet were very similar to changes in LDL cholesterol, and consequently imply an increased cardiovascular risk.

The LCHF diet induced a rather modest increase in HDL cholesterol (effect size 0.32 mM) in the current trial. Epidemiological data show that low HDL cholesterol is strongly associated with high risk of CVD [46], and measurement of HDL cholesterol seems useful for hyperlipidemia diagnosis and risk estimation [47,48,49]. Nonetheless, accumulating evidence over the last two decades indicates that low HDL cholesterol is unlikely to be a causal factor for atherosclerotic CVD, but instead a long-term marker of high levels of triglyceride-rich lipoproteins [50]. It is possible that other HDL parameters (such as function and cholesterol efflux) may be better markers of CVD risk [51], but this requires further development of HDL quality assays and clinical validation.

The LCHF diet induced a small but statistically significant increase in TG concentration (effect size 0.13 mM) in the current trial. This is in contrast to reports of normal weight, healthy, non-energy restricted men where a LCHF diet induces a decrease in TG [36,37,38]. In studies where both men and women participated, TG did not change [36,37,38]. This suggests there may be sex-specific differences in TG response following a LCHF diet. Plasma levels of TG are a well-established independent biomarker of CVD risk [52], and TG-rich lipoproteins have atherogenic properties. However, since TG can be degraded by the body, TG themselves will not accumulate in the atherosclerotic plaque. Thus, it has been suggested that high TG levels should only be viewed as a marker of high concentrations of cholesterol in TG-rich lipoproteins, i.e., remnant cholesterol [50].

The small sample size of the current trial is a limitation. However, the statistical power of a crossover study is much greater than in a parallel study of equivalent size since all participants receive the treatment under investigation [53]. In this trial, seven dropouts occurred on the ketogenic diet and this may introduce a possible bias into the results. Another potential limitation of our current trial is the somewhat greater weight loss induced by LCHF compared with the control diet. Although the participants were instructed to increase their food intake if losing weight, these instructions did not prove to be sufficient. However, reanalyzing the data and adjusting for relative weight loss did not change the results. If anything, weight loss is expected to elicit beneficial effects on blood lipids and CVD risk. Finally, this four-week feeding trial is obviously too short, and was not designed to see episodes in diet-related diseases like diabetes and CVD.

## 5. Conclusions

The current trial shows that feeding healthy, young women a ketogenic LCHF diet rich in saturated fatty acids for four weeks results in profound alterations in the blood lipid profile. In comparison with a control diet recommended by the Swedish National Food Agency, eating LCHF induced an increase in LDL cholesterol, a leading cause of atherosclerosis. LDL cholesterol increased in every participant with a treatment effect of 1.82 mM. We found an increase in both small, dense LDL cholesterol as well as large, buoyant LDL cholesterol. We also report an increase in ApoB indicating an increase in the total number of atherogenic lipoproteins in the circulation. These alterations should be a cause for concern in young, normal-weight, healthy women following this kind of LCHF diet.

## Figures and Tables

**Table 1:** Subject characteristics ^1,2^

Parameters	Median	(min–max)
Age (year)	23.8	(19.7–27.3)
Body weight (kg)	60.8	(50.8–70.9)
BMI (kg/m^2^)	22.0	(19.4–24.0)
Waist (cm)	71.0	(67.0–78.0)
Hip (cm)	97.0	(87.5–100.0)
WHR	0.76	(0.70–0.81)
Systolic BP (mmHg)	106	(89–118)

^1^ This table has been adapted from [27]. ^2^ Nine subjects used contraceptive hormones. BMI, body mass index; BP, blood pressure; WHR, waist/hip ratio. *n* = 17.

**Table 2:** Baseline measures (collected 2 to 5 days before starting the first diet intervention) and treatment effects of a four-week ketogenic LCHF diet ^1^.

Parameters	Baseline (Mean ± SD)	Treatment Effect (95% CI)	*p* Value
Primary outcome, mM			
LDL cholesterol	2.1 ± 0.6	1.82 [1.24, 2.39]	<0.001
LDL subfractions, mg/dL			
LDL 1–2 (large, buoyant LDL)	42.1 ± 14.6	31.56 [21.60, 41.51]	<0.001
LDL 3–7 (small, dense LDL)	2.7 ± 2.5	4.51 [1.87, 7.16]	<0.01
LDL particle size, nm			
LDL size	270 ± 3	−1.40 [−3.10, 0.30]	0.30
Blood Biochemistry, mM			
Glucose	4.9 ± 0.2	−0.49 [−0.68, −0.29]	<0.001
Insulin	6.3 ± 1.6	−2.94 [−4.00, −1.88]	<0.001
Standard chemical lipids, mM			
TG	0.6 ± 0.3	0.13 [0.05, 0.21]	<0.01
TC	4.1 ± 0.9	2.25 [1.65, 2.84]	<0.001
HDL cholesterol	1.7 ± 0.5	0.32 [0.23, 0.40]	<0.001
Non-HDL cholesterol	2.4 ± 0.6	1.91 [1.34, 2.49]	<0.001
Apolipoproteins, g/L			
ApoB	0.70 ± 0.15	0.50 [0.35, 0.65]	<0.001
ApoA-I	1.56 ± 0.34	0.37 [0.30, 0.44]	<0.001
Ratios			
Total cholesterol/HDL	2.4 ± 0.5	0.82 [0.50, 1.14]	<0.001
ApoB/ApoA-I	0.46 ± 0.12	0.20 [0.12, 0.28]	<0.001
LDL/HDL	1.3 ± 0.4	0.78 [0.47, 1.10]	<0.001

^1^ The treatment effect is statistically significant when *p* < 0.05. Data were analyzed using a mixed model. SD, standard deviation; CI, confidence interval; LCHF, low-carbohydrate high-fat; LDL, low-density lipoprotein; TG, triacylglycerol; TC, total cholesterol; HDL, high-density lipoprotein; ApoB, Apolipoprotein B-100; ApoA-I, Apolipoprotein A-I. *n* = 17.

## Data Availability

The data presented in this study are available on request from the corresponding author.

## Supplementary Materials

The following are available online at https://www.mdpi.com/2072-6643/13/3/814/s1, Figure S1: Daily levels of ketone bodies in urine, Table S1: Treatment effects of a four-week ketogenic LCHF diet adjusted for relative weight change.
