# Supplementary material for: A Ketogenic Low-Carbohydrate High-Fat Diet Increases LDL Cholesterol in Healthy, Young, Normal-Weight Women: A Randomized Controlled Feeding Trial
Source: Nutrients. 2021 Mar 2;13(3):814. doi: 10.3390/nu13030814 (PMC8001988; doi:10.3390/nu13030814)
Supplement: Supplementary file 1 [file nutrients-13-00814-s001.pdf]

Supplemental Figure S1. Daily ketone bodies in urine

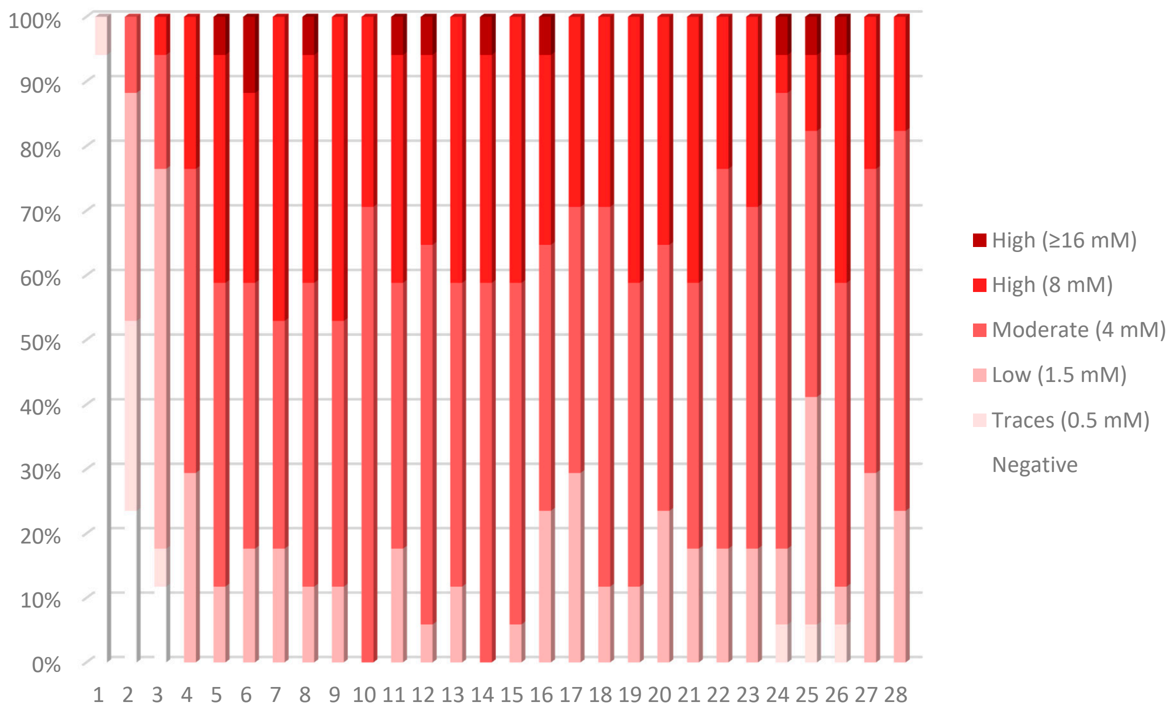

Daily urinary ketones in participants (*n* = 17) eating a ketogenic LCHF diet (Day 1-28, x-axis). Each bar (100%, y-axis) corresponds to all 17 participants.

Supplemental Table S1. Treatment effects of a four-week ketogenic LCHF diet adjusted for relative weight change[1]

**Parameters**	**Treatment effect (95% CI)**	***p* value**
Primary outcome, mM		
LDL cholesterol	1.83 [0.83, 2.84]	<0.01
LDL subfractions, mg/dL		
LDL 1-2 (large, buoyant LDL)	34.97 [18.07, 51.88]	<0.001
LDL 3-7 (small, dense LDL)	6.09 [1.50, 10.69]	<0.05
LDL particle size, nm		
LDL size	–1.72 [–4.69, 1.24]	0.23
Blood Biochemistry, mM		
Glucose	–0.31 [–0.64, 0.01]	0.059
Insulin	–2.64 [–4.55, –0.73]	<0.05
Standard chemical lipids, mM		
TG	0.16 [0.03, 0.29]	<0.05
TC	2.26 [1.21, 3.31]	<0.001
HDL cholesterol	2.26 [1.21, 3.31]	<0.001
Non-HDL cholesterol	1.98 [0.98, 2.98]	<0.001
Apolipoproteins, g/L		
ApoB	0.52 [0.26, 0.77]	<0.001
ApoA-I	0.40 [0.28, 0.53]	<0.001
Ratios		
Total cholesterol/HDL	0.93 [0.39, 1.46]	<0.01
ApoB/ApoA-I	0.19 [0.05, 0.33]	<0.05
LDL/HDL	0.85 [0.31, 1.39]	<0.01

[1]The treatment effect is statistically significant when $p < 0.05$. Data were analyzed using a mixed model, with post measurements of each period as the dependent variable. Period baseline measurements, and diet were included as fixed effects and subject was included as a random effect. Subject-averaged period baseline values were included as fixed covariates, to avoid cross-level bias. LCHF, Low-carbohydrate high-fat; TG, Triacylglycerol; TC, Total cholesterol; ApoB, Apolipoprotein B-100; ApoA-I, Apolipoprotein A-I.
